# Macroevolutionary integration of phenotypes within and across ant worker castes

**DOI:** 10.1002/ece3.6623

**Published:** 2020-08-18

**Authors:** Nicholas R. Friedman, Beatrice Lecroq Bennet, Georg Fischer, Eli M. Sarnat, Jen‐Pan Huang, L. Lacey Knowles Knowles, Evan P. Economo

**Affiliations:** ^1^ Biodiversity and Biocomplexity Unit Okinawa Institute of Science and Technology Graduate University Okinawa Japan; ^2^ Department of Ecology and Evolutionary Biology, Museum of Zoology University of Michigan Ann Arbor MI USA; ^3^ Biodiversity Research Center Academia Sinica Taipei Taiwan

**Keywords:** ants, caste, dimorphism, geometric morphometrics, modularity, morphological integration, *Pheidole*

## Abstract

Phenotypic traits are often integrated into evolutionary modules: sets of organismal parts that evolve together. In social insect colonies, the concepts of integration and modularity apply to sets of traits both within and among functionally and phenotypically differentiated castes. On macroevolutionary timescales, patterns of integration and modularity within and across castes can be clues to the selective and ecological factors shaping their evolution and diversification. We develop a set of hypotheses describing contrasting patterns of worker integration and apply this framework in a broad (246 species) comparative analysis of major and minor worker evolution in the hyperdiverse ant genus *Pheidole*. Using geometric morphometrics in a phylogenetic framework, we inferred fast and tightly integrated evolution of mesosoma shape between major and minor workers, but slower and more independent evolution of head shape between the two worker castes. Thus, *Pheidole* workers are evolving as a mixture of intracaste and intercaste integration and rate heterogeneity. The decoupling of homologous traits across worker castes may represent an important process facilitating the rise of social complexity.

## Graphical abstract

Here, we test the extent to which morphological traits in different social insect worker castes should exhibit integration and modularity. We used geometric morphometric data on major and minor workers' head and thorax shape from 246 species in a phylogenetic comparative analysis of evolutionary integration and rate. Our results show that thorax shape evolved in a rapid and tightly integrated fashion, whereas head shape evolved slower and more independently.

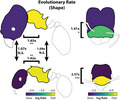

## 1 INTRODUCTION

The increase of morphological complexity following divergence in cellular function is a repeating theme in the evolution of multicellular organisms (Wagner & Altenberg, 1996). Given cues regarding their developmental fate, cells and tissues express their identical genomes in different ways to produce different traits and thus allow functional specialization. Morphological integration can be considered the extent to which these traits vary in concert, either as a continuation of their shared genetic or developmental origin, or as a unification of parts contributing to a shared function and shaped by selection (Klingenberg, 2008; Olson & Miller, 1958). Sets of integrated traits covary as modules, between which covariation is weaker than within (as in the primate cranium; Cheverud, 1982).

Much as a single genome can underlie different cooperating tissues and traits within the same organism, different traits are also produced among individuals using the same genome. Distinct phenotypes are commonly observed in different sexes (Owens & Hartley, 1998), or in individuals adopting alternative reproductive tactics (Emlen, Lavine, & Ewen‐Campen, 2007) as a result of differential selection. Eusocial insects reflect a major evolutionary transition whereby a unit of selection is comprised of different individuals working together as part of an integrated colony‐level phenotype (Hölldobler & Wilson, 1990; Szathmáry & Smith, 1995; Wheeler, 1911) and understanding the evolution and function of these “superorganisms” is a major and enduring interest of evolutionary biology (Hölldobler & Wilson, 2009; Oster & Wilson, 1978; Seeley, 1995). The castes of social insects can exhibit radically different traits from the same genome: a female egg laid by the queen has the potential to develop into either another queen or a worker caste individual. This phenotypic polymorphism allows functional specialization among individuals in a colony and the rise of social complexity, the feature of eusociality that best defines its potential for division of labor (Hölldobler & Wilson, 1990; Oster & Wilson, 1978). While worker castes are an ancestral trait shared by nearly all extant ants, several lineages have since evolved further division of labor among workers to form worker castes—known also as subcastes (Hölldobler & Wilson, 1990; Oster & Wilson, 1978; Wills, Powell, Rivera, & Suarez, 2018; Wilson, 1953; Fjerdingstad & Crozier, 2006). In the colonies of some species (e.g., *Solenopsis invicta*), worker castes exhibit polymorphism mostly along a single allometric function—shape varies with size along a regular continuum (Wilson, 1953). However, for species in other genera (e.g., *Pheidole*, *Colobopsis*, *Carebara*, *Cephalotes*, *Eciton*, *Acanthomyrmex*, *Pseudolasius*), variation reaches “complete dimorphism” into distinct major worker and minor worker phenotypes (Wilson, 1953). While there is some contention over nomenclature within the myrmecological community (Urbani, 2015), we refer to minor workers and major workers (aka soldiers) as “worker castes” throughout following Wills et al. (2018).

The evolution of complete dimorphism offers the potential for new dimensions of variation in ants (Wills et al., 2018; Wilson, 1953). If phenotypes are disintegrated among worker castes, this can allow for greater functional specialization and different combinations of traits available to the colony‐level phenotype (Powell, 2008, 2009; Wills et al., 2018; Wilson, 1953). However, the evolution of specialized morphology in major workers may be biased by developmental pathways that are shared with minors (Rajakumar et al., 2012; Wheeler, 1991; Wheeler & Nijhout, 1983, 1984); thus, there could be limits to divergence among homologous body parts across the different worker castes, or a shared pathway could lead selection on one worker caste to result in a neutral change in the other.

The ecological and behavioral roles of polymorphic worker ants have long been a fascination of social insect research (Goetsch, 1937; Oster & Wilson, 1978; Powell, 2008, 2009, 2016; Powell & Franks, 2006; Wheeler, 1911; Wills et al., 2018; Wilson, 1953). Likewise, the genomic and biochemical mechanisms underpinning caste differentiation are a central avenue for understanding the evolution of social complexity (Anderson et al., 2008; Chandra et al., 2018; Gospocic et al., 2017; Hughes et al.,&^2003^; Lillico‐Ouachour & Abouheif, 2016; Molet et al., 2012; Rajakumar et al., 2012; Wheeler, 1991). However, the macroevolutionary implications of these processes—the patterns of integration and modularity that emerge across the diversification of hundreds or thousands of lineages—are less well studied in ants than other taxa, although the topic is receiving increasing interest (Holley, Moreau, Laird, & Suarez, 2016; Pie & Traniello, 2007; Pie & Tschá, 2013; Powell, 2016). These patterns, revealing the degree to which different traits evolve independently within and among worker castes, may be an important clue to both the selective forces driving evolution like ecological subspecialization among worker castes (Powell & Franks, 2006), and the potential constraints on evolution like restrictive developmental limitations (as in Fritz et al., 2014). Furthermore, the differences in evolutionary rates among different traits in the same worker castes, or the same trait in different worker castes, may reflect aspects of the phenotype that are under strong selection because they underlie axes of ecological divergence among species (Price, Etienne, & Powell, 2016; Schluter, 2000).

Ants have colonized and evolved adaptations to many environments, and are among the most abundant terrestrial organisms on the planet. Ants have also radiated to produce a diverse array of morphologies in nearly every region they have colonized (Hölldobler & Wilson, 1990). In this study, we focus on overall body size, the relative body sizes of different parts, and the shapes of the head and mesosoma. Body size has been shown to be a major axis of morphological variation among ants (Pie & Traniello, 2007; Powell, 2016; Price et al., 2016). Previous studies of functional morphology in ants have also focused on the head (e.g., Holley et al., 2016), which contains the main apparatus for feeding (mouthparts, mandibles), manipulating objects (mandibles), and sensation (eyes, antennae). If head shape is the primary focus of ecological adaptation, this trait should evolve more rapidly than others during radiation. We also examine the mesosoma, the main power center of the ant including muscles for bearing loads and moving the legs. While the functional significance of external mesosoma shape is not well understood, the shapes and relative sizes of different regions likely reflect investment in different muscle groups that have functional implications. The sizes and positions of the sclerites (plates of the cuticle which are partially captured here by our landmarks) are associated with homologous attachment points underneath. For example, Keller, Peeters, and Beldade (2014) showed that the pronotal region associated with the T1 sclerite houses the muscles that lift the head. Other regions of the mesosoma contain stabilizing muscles, muscles to support the legs, and muscles to flex the petiole (Lubbock, 1881), all of which have obvious functional implications.

Relatively, few studies have compared the tempo of evolution across different ant traits, (but see Blanchard & Moreau, 2017; Holley et al., 2016; Pie & Tschá, 2013). If the shapes of other traits such as the mesosoma (thorax) evolve more rapidly, this may be an indication that they serve a greater functional role in ecological divergence than previously understood. Likewise, if majors exhibit greater rates of change than minor workers, that may signal that their functional role has changed often following the evolution of complete dimorphism or that they are important for achieving and maintaining ecological divergence among species.

To compare morphological integration and evolutionary rate of different worker castes and traits, we focused on the ant genus *Pheidole*. The ants of this genus have, in the course of their approximately 37 million year history, spread throughout six continents to produce more than 1,000 described (and many more undescribed) species (Economo, Klimov, et al., 2015; Moreau, 2008). Perhaps the most notable characteristic of species in this hyperdiverse genus is the clear dimorphism of their workers: a major worker caste with enlarged heads is easily visible in all species (indeed a third supermajor form is also observed in some species) (Wilson, 2003). Behavioral studies have described different ecological roles for *Pheidole* worker castes, with major workers performing more defense, food processing, and storage tasks than minor workers (Huang, 2010; Mertl & Traniello, 2009; Tsuji, 1990; Wilson, 1984). The relatively consistent body plan and caste structure of this genus make it an ideal clade for comparative studies of morphology (Holley et al., 2016; Pie & Traniello, 2007). The developmental basis of worker caste differentiation in *Pheidole* has been well studied over the years (Lillico‐Ouachour & Abouheif, 2016; Rajakumar et al., 2012, 2018; Wheeler & Nijhout, 1983, 1984), and recent work on the taxonomy, biogeography, and ecomorphology of this group (Economo & Sarnat, 2012; Economo, Sarnat, et al., 2015; Holley et al., 2016; Mertl & Traniello, 2009; Muscedere & Traniello, 2012; Sarnat et al., 2017; Sarnat & Moreau, 2011; Wilson, 2003) make it an attractive model clade for evolutionary research on social insects.

Several previous studies on the macroevolution of *Pheidole* morphology are particularly relevant for the current investigation. First, in an analysis before a *Pheidole* phylogeny was available, Pie and Traniello (2007) analyzed morphology with linear measurements and found that size differences explained most of the variation in *Pheidole* morphology across species, but majors and minors showed divergent patterns of character correlation. Later, with the benefit of a *Pheidole* phylogeny (Moreau, 2008), Pie and Tscha (2013) showed that size varied more quickly than shape variables based on linear morphometrics, but did not explicitly test for modularity and integration. Holley et al. (2016) found that known ecological specialization of majors (seed milling behavior in granivorous species) was related to divergence in head size between major and minor worker castes (although enigmatically, due to a change in the minors), evidence that independent evolution of the two worker castes in relation to ecology can occur. Finally, Sarnat et al. (2017) tested hypotheses for the evolution of exaggerated thoracic spines, an unusual and geographically restricted phenotype in *Pheidole*.

Despite the insights of these pioneering studies, a comprehensive picture of the roles of integration, modularity, and rate heterogeneity in morphological evolution within and among *Pheidole* castes has not emerged. Using landmark‐based geometric morphometrics, and taking advantage of recent progress on reconstructing the *Pheidole* phylogeny (Economo et al., 2019; Economo, Klimov, et al., 2015) which allows for a more taxonomically and geographically extensive analysis, we perform the most morphologically and phylogenetically comprehensive analysis to date to attempt to infer a general picture of integration and modularity in size and shape in the *Pheidole* worker castes.

**FIGURE 1:**
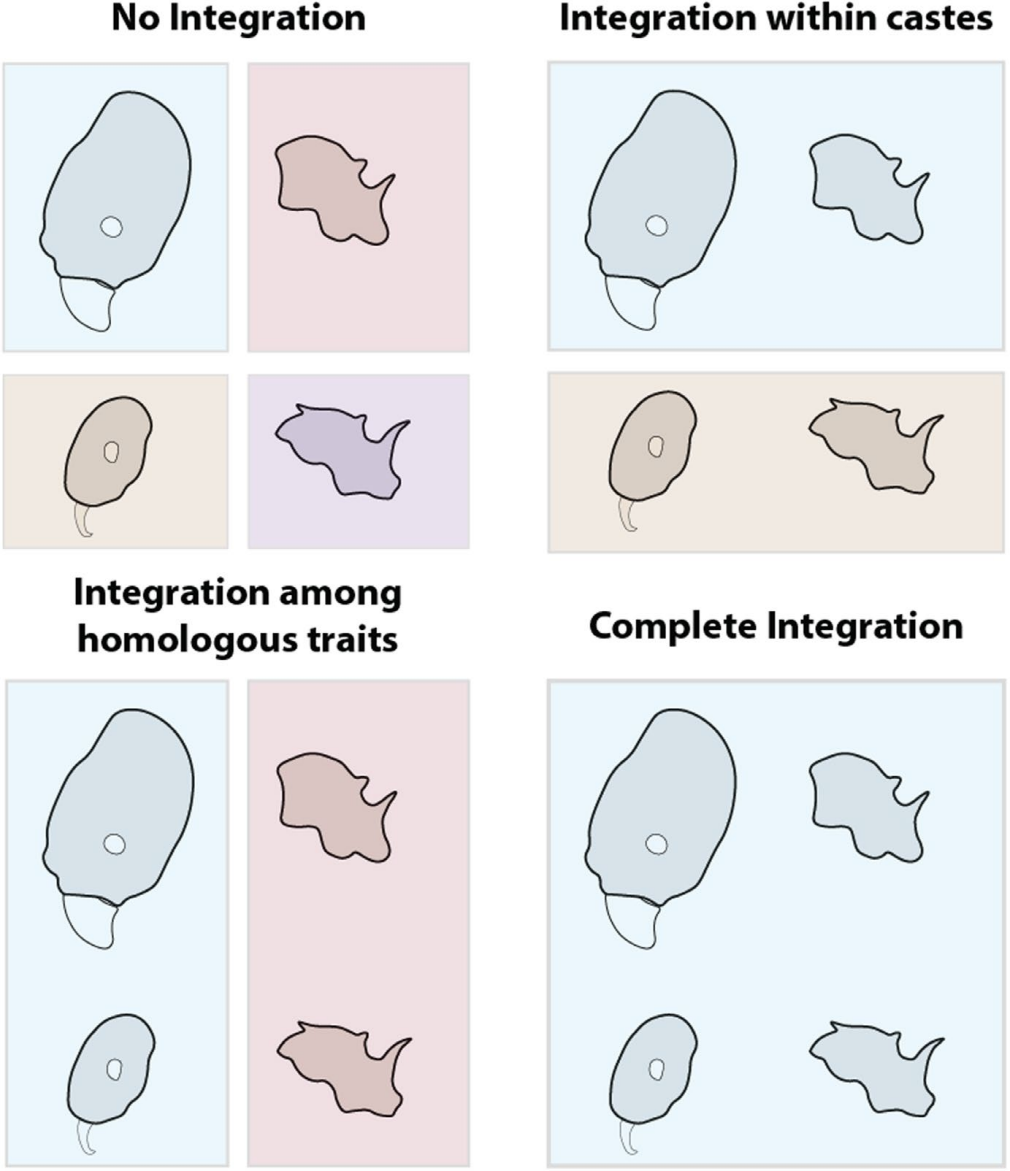
Hypothesized scenarios for the evolution of differentiated phenotypes. Worker castes or body parts united in the same box represent a pair of integrated traits. The scenarios we propose can be arranged in order of their extent of integration among homologous traits in different castes and among different traits within a caste

To frame our study, we propose a set of hypotheses predicting different patterns of morphological integration within and among castes in social insect colonies (see Figure 1). We discuss this in terms of the head and mesosoma (thorax) of *Pheidole* worker castes, but it could equally be applied to any morphological traits shared among castes, or indeed traits shared among other differentiated phenotypes like sexes or reproductive strategies (Simpson et al., 2011). First, different parts of the body *within* a worker caste may be more or less integrated. This integration could reflect developmental biases or biomechanical constraints; for example, a specific change in head morphology may necessitate a specific change of the thoracic segments that support or move the head. Second, *across* worker castes the same homologous body parts could be more or less integrated. As different worker castes share not only genomes but developmental pathways, it is plausible that selection on a trait in one worker caste could lead to a change in another worker caste. For example, selection on elongation of the head of a minor worker may lead to similar elongation in the major worker, even if there is no inclusive fitness benefit to the change in the major worker. Or, each worker caste could vary independently facilitating different functional roles in the colony.

We test these hypotheses by assessing the presence and pattern of integration of the head and mesosoma within and among worker castes. First, we assess heterogeneity in rates of evolution across body parts and worker castes; whether evolutionary change tends to follow a pattern in which different parts or worker castes are hot or cold spots of change, or whether traits evolve at similar rates within and among worker castes. Second, we look for patterns of modularity in shape and size to test how well an evolutionary change in shape or size of one trait predicts the shape and size of another trait within the same worker caste or in a different one. If there are differences in evolutionary rates, we ask again whether those differences reflect characteristics shared among homologous traits or among worker castes.

## 2 METHODS

### 2.1 Photographic measurements

All comparative studies reflect a compromise between depth of individual sampling within species versus breadth across species. In this study, we aimed to expand the latter to include as many *Pheidole* species as possible. We acknowledge a drawback of this strategy, which is that we cannot capture the size or shape range of individuals within each species. We measured a total of 1,164 specimens from 314 species, measuring an average of 2.18 major worker and 2.20 minor worker specimens per species; to maintain consistency between samples, all measurements were performed by coauthor BL. Myrmecologists use high‐resolution montage photographs to document ant diversity, following a standardized set of specimen positions that display head and body features from a consistent angle as described by the online resource and repository, AntWeb.org. We made a broad effort to photograph specimens from species used in recent phylogenetic projects (Economo, Klimov, et al., 2015), supplemented with photographs taken by others and deposited on Antweb.org. We endeavored to collect data on both major and minor workers whenever possible; however, photographic data for both worker castes were only available for 214 species or 68% of our total taxonomic sample. To account for potential focal length issues when using 2D photographs taken with different optical systems, we landmarked the same specimen 100 times under six different magnifications. A focal length warping effect was observable but was nonsignificant, and was within the range of intraspecific variation.

**FIGURE 2:**
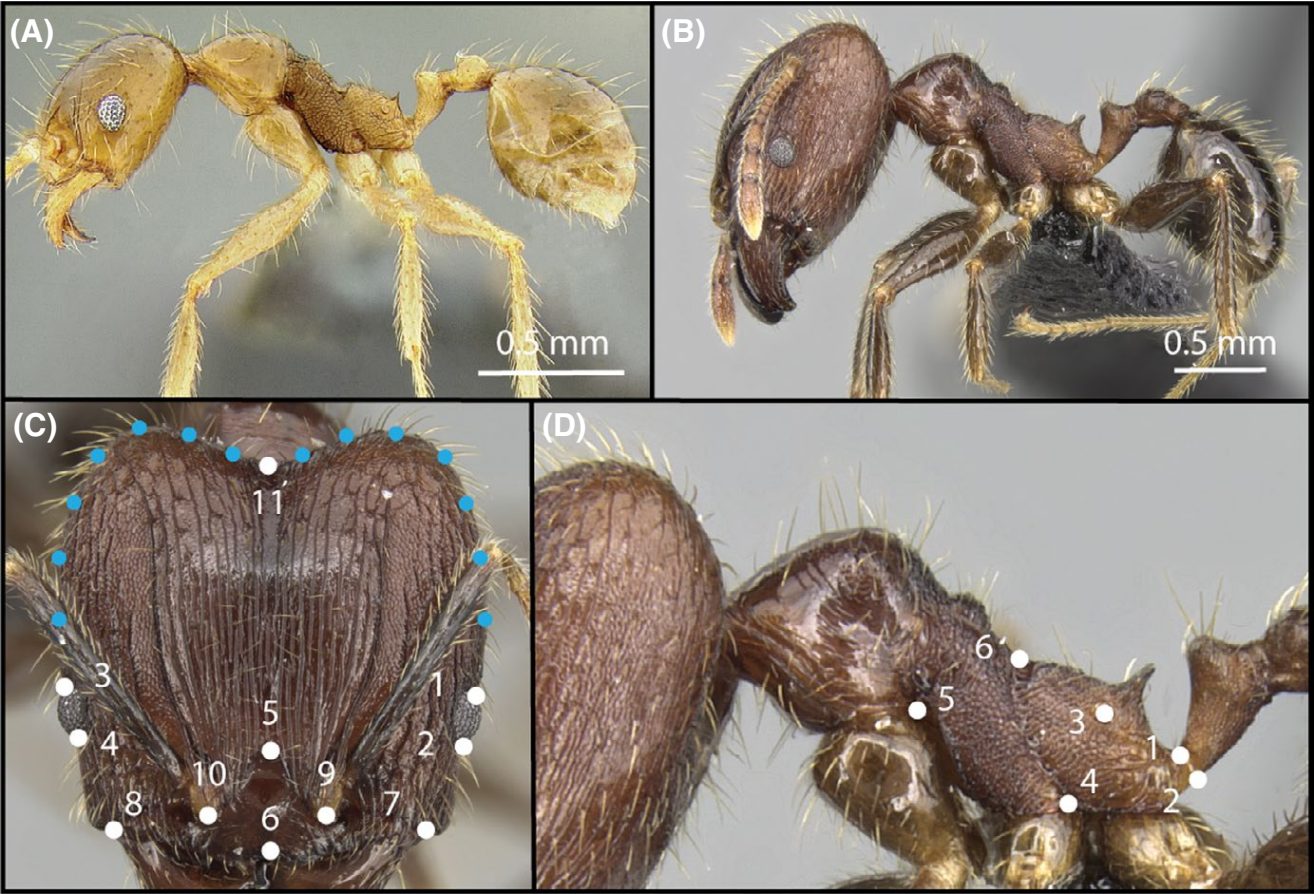
Example photographs of *P*. *fervens* minor worker (a) and major worker (b) assembled by photomontage according to AntWeb specifications. Landmarks, in white, were placed on homologous features on the head (c) and mesosoma (d). Semilandmarks, in blue, were spaced equally on the left side of the head between landmarks 3 and 11, and between landmarks 11 and 1

For each specimen, we placed landmarks using the three standard photographic angles: head view, dorsal view, and profile view. We collected landmarks from features that were consistently in the plane of the camera angle. Specifically, we placed 11 landmarks on the dorsal view of the head (Appendix S1) and six landmarks on the profile view of the body (Appendix S2; all located on the mesosoma; hereafter head, mesosoma; see Figure 2). To capture information on the posterior head shape, we also included a set of 6 sliding semilandmarks (7 in major workers) from landmark 3 to 11 (Figure 2). The landmarks on the left side of the head were reflected bilaterally to produce the curve on the right side of the head between landmarks 11 and 1. Fixed landmarks on opposite sides of the head were reflected and averaged to force object symmetry.

While these landmarks omit several features that vary among *Pheidole* taxa, and those typically used in myrmecology research and taxonomy (Pie & Traniello, 2007), this was unavoidable due to the constraints of choosing homologous landmarks in positions that are not occluded by nearby features (e.g., the anterior pronotum is often occluded by the posterior head lobes).

### 2.2 Geometric morphometrics

**FIGURE 3:**
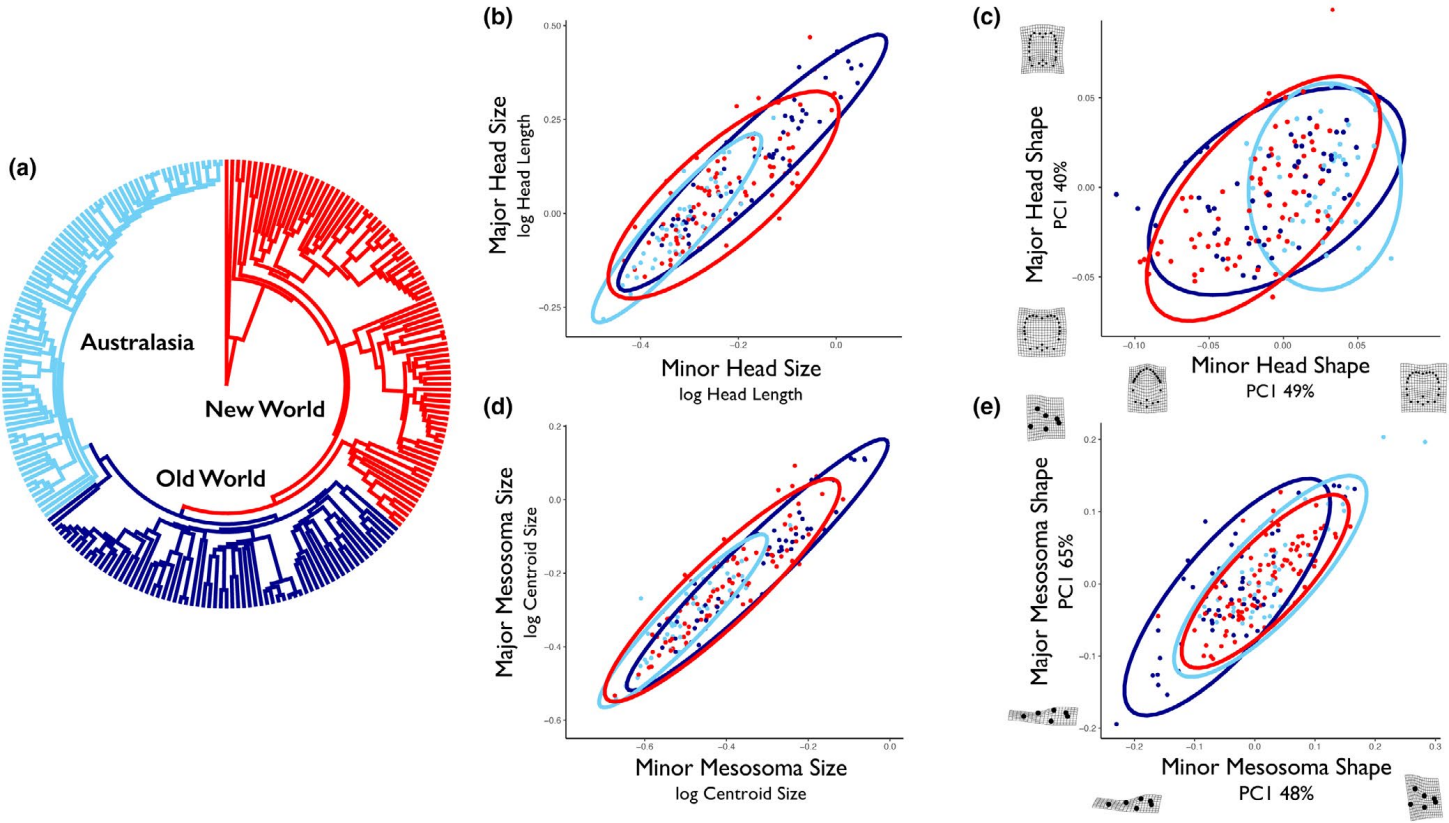
A phylogeny of the ant genus *Pheidole*, with clades colored by their geographic region, is shown in (a). Note that each clade represents a single colonization event (see Economo, Klimov, et al., 2015). Comparisons of values for like traits in different castes are shown for head size (b), head shape (c), mesosoma size (d), and mesosoma shape (e). Ellipses reflect 95% confidence intervals and are colored according to clade as in (a). For the shape data displayed in (c) and (e), the first principle component is shown for display purposes (and is not used in subsequent comparative methods), along with the percentage of variance it explains and deformation grids describing extreme values along the axis (produced using *geomorph*; Adams & Collyer, 2018)

We performed a generalized Procrustes alignment on each set of landmarks using the R package *geomorph*, employing separate analyses for major and minor workers (Adams & Otárola‐Castillo, 2013; version 3.0.7). Specimens showing greater than expected distance from the Procrustes mean (i.e., above the upper quartile) were inspected for improper scale entry or landmark order/placement. Photographs for which improper specimen positioning was observed were removed from the dataset (<1% of specimens studied). Within each species, we calculated the average Procrustes shape before proceeding with further analyses; we also averaged linear measurements in this manner. To visualize variation in highly dimensional shape characters, we estimated principal component axes and plotted species averages in tangent space (Figure 3c,e). As a proxy for body size, we used the logarithm of the centroid size of mesosoma landmarks as in (Economo, Klimov, et al., 2015), which behaves similarly to the Weber's Length measurement typically used by myrmecologists (Weber, 1938). Only multivariate Procrustes alignment data, and not principle component data, were used in the comparative methods below (Uyeda, Caetano, & Pennell, 2015).

### 2.3 Phylogenetic data

We used a time‐resolved phylogeny reconstructed by Economo, Narula, Friedman, Weiser, and Guénard (2018) that includes 449 ingroup *Pheidole* species, based on a molecular dataset of nine loci. This phylogenetic tree builds upon previous analyses of *Pheidole* (Economo, Klimov, et al., 2015; Moreau, 2008), with the addition of 164 taxa and an expanded set of loci sequenced across species. For analyses in this paper, we used the maximum clade credibility tree from a Bayesian posterior set, which was pruned to contain only the taxa present in our morphological data (Figure 3a).

### 2.4 Comparative methods

To examine the degree of correlated evolution between body regions (i.e., morphological integration), we used the *R* package *geomorph* (Adams & Otárola‐Castillo, 2013). We ran a series of pairwise integration tests between body regions and worker castes (Adams & Collyer, 2017). In each test, we estimated partial least squares (PLS) correlations between two sets of landmarks (e.g., major's head and minor's head) while correcting for phylogeny. The coefficient of correlation (r‐PLS) for this regression describes the degree of integration. To calculate a p‐value and significance test, we generated 1,000 permutations of species’ phylogenetically transformed values for each comparison. To compare evolutionary integration of body size among worker castes and between the head and mesosoma, we used the coefficient of correlation for the linear regression of phylogenetically independent contrasts, hereafter r‐PIC (Felsenstein, 1985). To compare the relationship between trait shape and body size, we used a phylogenetic regression implemented for Procrustes shape variables (Adams & Collyer, 2018). These and other methods described below were also run for the New World, Old World, and Australasian clades individually (Figure 3a). It is important to note that integration may exceed the values estimated here using PLS, as integration may span multiple PLS axis dimensions beyond the first axis, which is what we compared.

We used *geomorph* to estimate evolutionary rates for landmarked specimens (Denton & Adams, 2015). As a significance test for differences in rates between traits, we performed 1,000 simulations of trait evolution under a joint Brownian motion model and compared the ratio of independently estimated rates to this simulated null. Given that differences in the number of landmarks can bias the amount of variation and thus rate described by each trait (Denton & Adams, 2015), we report rate ratios for each pair of traits (e.g., major head vs. major mesosoma) as a proportion of the simulated null ratio.

**FIGURE 4:**
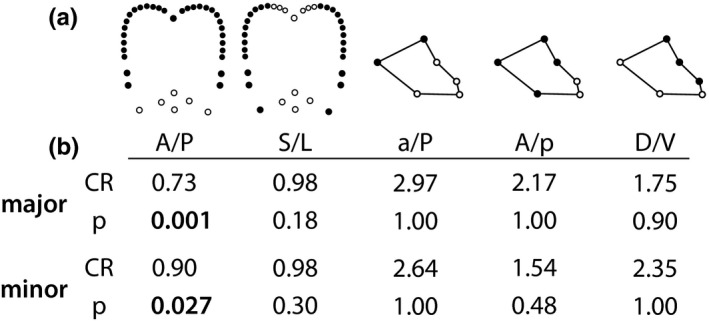
Hypothesized evolutionary module configurations (a) are shown with landmarks assigned to each module assigned with different colors. The table in (b) shows results of phylogenetically corrected modularity tests conducted in geomorph. Covariance ratios (CR) are given for each hypothesized configuration, as well as p‐values derived from comparison against a simulated null

We tested for evidence of evolutionary modularity within each body region (i.e., in addition to the head and mesosoma) again using *geomorph* (Adams & Otárola‐Castillo, 2013). We split each body region into sets of a priori evolutionary modules (sensu Klingenberg, 2008) roughly aligned with anatomical axes. Head landmarks were assigned to two potential module arrangements: one along the anterior/posterior axis (hereafter: A/P), and one along the sagittal/lateral axis (hereafter: S/L; see Figure 4a). The A/P grouping separates the anterior (clypeus) area which is related to the feeding apparatus from the posterior of the head which houses the brain and mandible muscles. The D/V axis separates structures more toward the midline of the head (central clypeus, antennae) from the sides (eyes, occipital lobes). Mesosoma landmarks were also assigned to three potential groupings: one along the anterior/posterior axis with bias toward the anterior (hereafter: A/p), one along a similar axis with bias toward the posterior (a/P), and one along the dorsal/ventral axis (D/V; see Figure 4a). These anterior/posterior groupings correspond to landmarks associated with different body segments, while the D/V grouping associates landmarks in the region closer the legs or dorsal part of the body, respectively. In this framework, we compared the covariance ratio (CR; Adams, 2016) of each hypothesized set of landmarks to those of simulated sets of landmarks (averaged between orientations rotated up to 90° in 0.05° increments), while accounting for phylogenetic relationships. Each simulation test was run for 1,000 iterations.

## 3 RESULTS

### 3.1 Evolutionary rate

**FIGURE 5:**
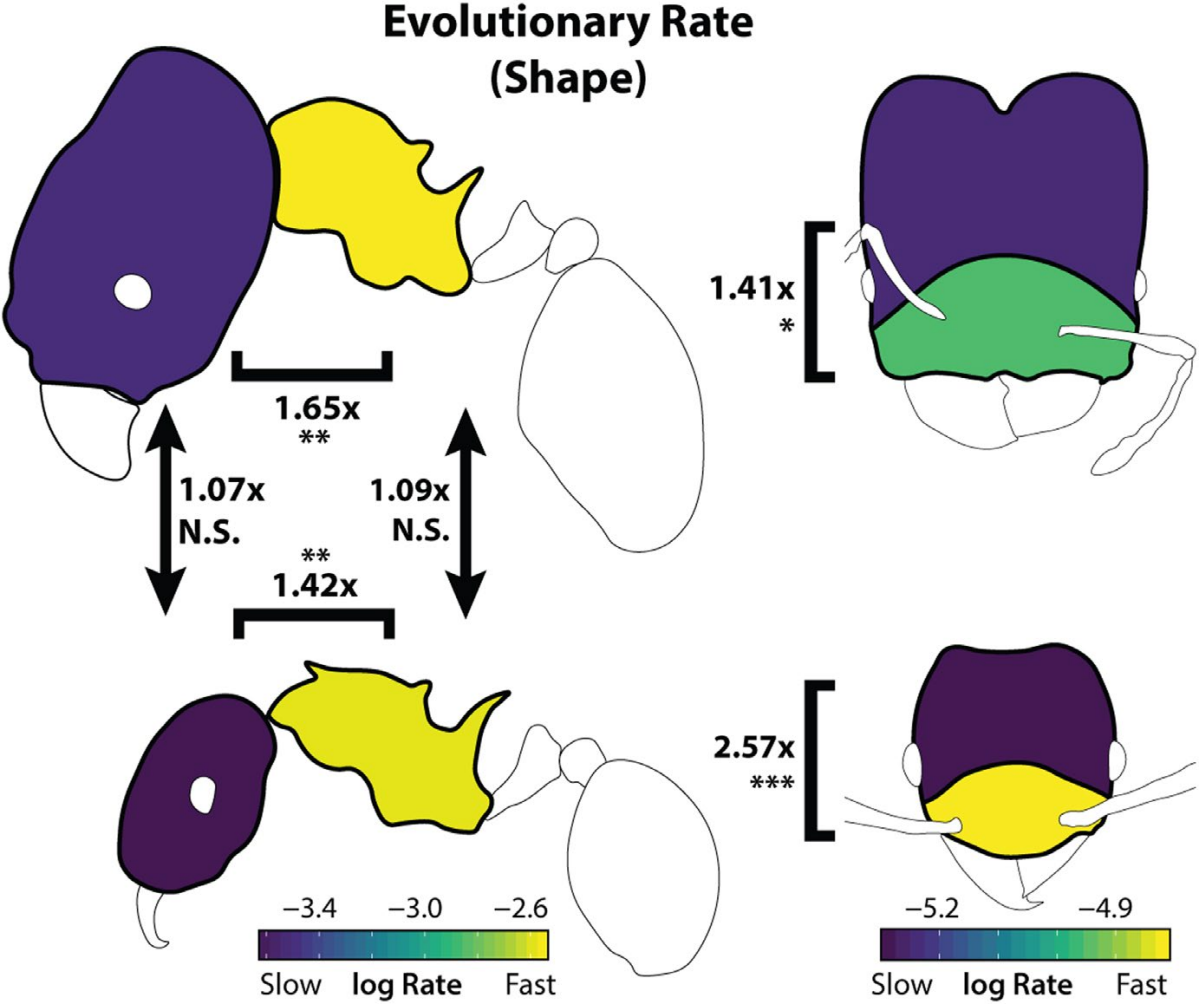
Evolutionary rates are displayed here as a morphogram heat map (Martin & Wainwright, 2011). Comparison ratios between traits digitized using different numbers of landmarks (e.g., head and mesosoma) are given as ratios compared to a simulated null ratio. Arrows and brackets indicate statistical tests of rate differences compared to a simulated null, with accompanying numbers describing the estimated rate ratio for the two traits. *p < .05, **p < .01, ***p < .001

**FIGURE 6:**
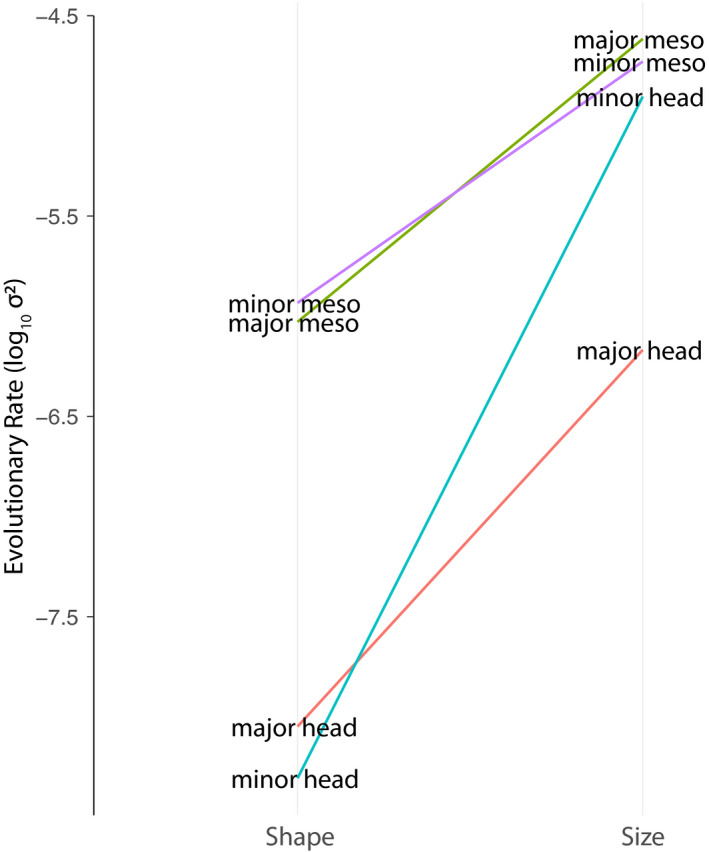
Comparison of evolutionary rate estimates for size and shape of *Pheidole* body parts and worker castes

In comparisons of different body regions of the same worker caste, mesosoma shape evolved more rapidly than head shape in both major workers (rate ratio rr = 6.02, *p* < .01) and minor workers (rr = 6.14, *p* < .01; Figure 5). In comparisons of similar traits between worker castes, we observed no significant differences in evolutionary rate for head shape (rr = 1.07, *p* = .59) or mesosoma shape (rr = 1.10, *p* = .59). In contrast with the rate variation among shape traits, evolutionary rates estimated for size traits showed few differences between worker castes or between the head and mesosoma (Figure 6), with the exception of the major worker's head which evolved relatively slowly.

Our tests of modularity within body regions suggested the presence of two evolutionary modules in the *Pheidole* head, in an anterior–posterior arrangement, though the use of semilandmarks may bias this result. We compared evolutionary rate between the inferred modules of head shape (Figure 4a). In these analyses, the anterior landmarks exhibited a higher rate of evolution than the posterior landmarks in both major workers (rr = 1.42, *p* < .05) and minor workers (rr = 1.42, *p* < .001).

### 3.2 Morphological integration

Morphological integration is described here as correlated evolution between morphological shape characters. The strength of this correlation is described using the PLS correlation coefficient (r‐PLS), and its significance is assessed by comparison to a simulated null distribution (Adams & Collyer, 2016; Adams & Felice, 2014). For estimates of body size rather than shape, it is measured as the correlation coefficient of independent contrasts (r‐PIC).

**FIGURE 7:**
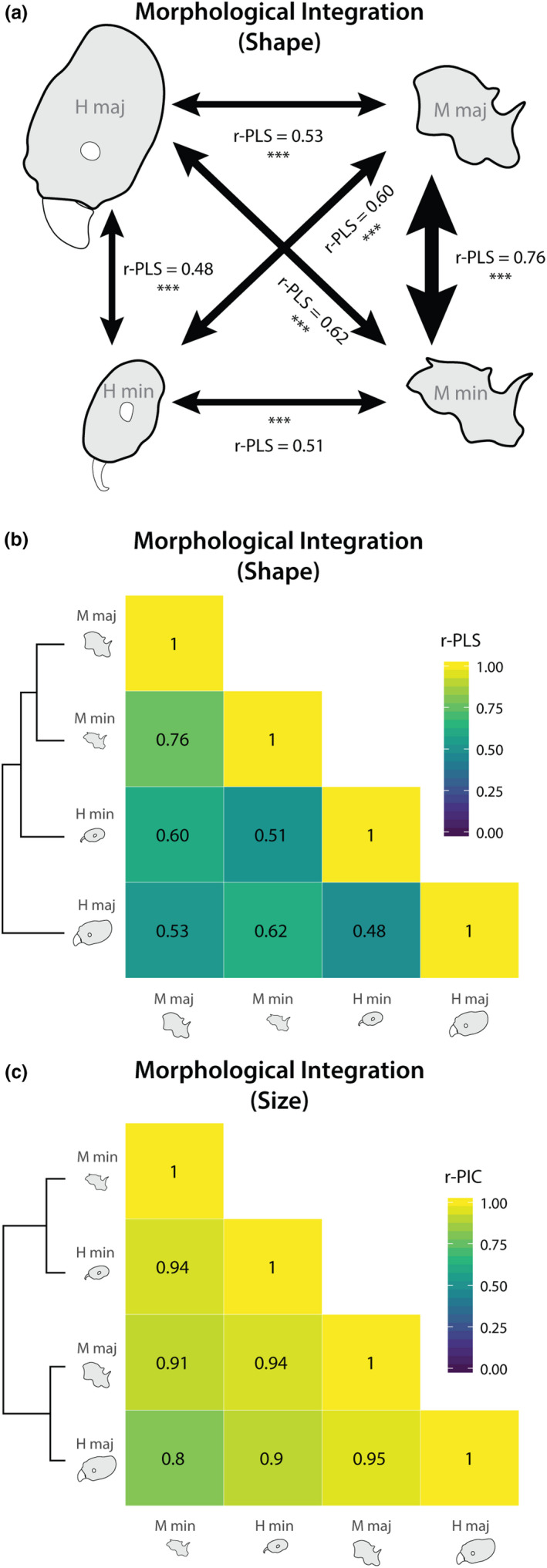
Morphological integration between among body parts within and among worker castes is shown by arrow width in (a). Hierarchical clustering of integration relationships for trait shape is shown in (b) and for trait size in (c), with the strength of relationships indicated by the heat map and displayed value—r‐PLS for trait shape and r‐PIC for trait size. *p < .05, **p < .01, ***p < .001

We found strong indications of morphological integration between both worker castes and body regions in *Pheidole;* however, the strength of these correlations varied depending on the comparison (Figure 7a). Head shape was correlated with mesosoma shape in both major workers (r‐PLS = 0.53, *p* < .001) and minor workers (r‐PLS = 0.51, *p* < .001). In examinations of morphological integration between worker castes, mesosoma shape was strongly correlated between castes (r‐PLS = 0.76, *p* < .001), whereas head shape showed a weaker albeit still significant correlation (r‐PLS = 0.48, *p* < .001). This difference in worker caste integration effect among body regions was highly significant (two‐sample z test; *p* < .001). Similar results were observed for analyses performed with semilandmarks from the head's posterior lateral lobes included. Morphological integration varied somewhat between clades, with the Asian‐African clade exhibiting a lower degree of integration for all shape traits.

We performed hierarchical clustering on correlation coefficient matrices for shape integration and size integration (Figure 7b,c). Overall, *Pheidole* showed much greater morphological integration in size than in shape. Morphological integration was greater for size traits (r‐PIC 0.8–0.95) than for any shape traits (maximum r‐PLS = 0.76). This integration in size was greater within worker castes than between them (Figure 7c). Morphological integration of shape traits was greatest between the mesosoma of major and minor workers, which evolved as though it were a single module. Head shape was weakly integrated with other traits for minor workers, and least integrated for major workers (Figure 7b).

The scaling relationship between the sizes of different parts is a common theme in evolution and development. As expected, we found a tight relationship between mesosoma size (Weber's length; Weber, 1938) and head length; this was evident in both majors and minors. Relationships between the shape of the head and mesosoma and body size were observable; however, they were very weak and poorly predictive (all *R*‐squared values < 0.03).

### 3.3 Modularity within body regions

Modularity is measured in geomorph as the covariance ratio (CR), which describes the covariation between modules relative to the covariation within modules (Adams, 2016). When CR ≥ 1, modules show no observable signal of modularity; values significantly less than 1 (compared to a simulated null distribution) indicate independence between modules. We estimated modularity for two a priori configurations for head landmarks and three a priori configurations for mesosoma landmarks (Figure 4a).

We tested for modularity in head shape using two datasets: one including only fixed landmarks, and one also including semilandmarks describing the curvature of the posterior lateral lobes. Using only fixed landmarks, head shape showed no significant modularity along the A/P axis in major workers (CR = 1.31, *p* = 1.00; see also Figure 4b) or minor workers (CR = 1.28, *p* = 1.00). We observed similarly nonsignificant scores when dividing landmarks into a S/L axis; this was consistent across both major workers (CR = 1.17, *p* = .91) and minor workers (CR = 1.18, *p* = .87). However, when we included (semilandmark) data on the posterior lateral lobes, we observed that head shape evolved as two independent modules along the anterior/posterior axis in major workers (CR = 0.79, *p* = .003) and in minor workers (CR = 0.76, *p* = .002). No such effect was observed along the sagittal/lateral axis for either major workers (CR = 0.98, *p* = .26) or minor workers (CR = 1.05, *p* = .69). For mesosoma shape, we observed no significant or even weak evidence of modularity (always CR > 1; see Figure 4b). However, few potential module configurations exist for a set of only 6 landmarks, which likely limited our ability to detect modularity with this dataset.

## 4 DISCUSSION

Our results showed varying evolutionary rates and degrees of evolutionary integration within and among worker castes; thus, evolutionary rate and integration followed the predictions of different hypotheses (Figure 1). In particular, the mesosoma exhibited integration among homologous traits in different worker castes, while the head exhibited a weaker degree of integration. We found that the mesosoma evolved faster than the head and with a greater degree of morphological integration between castes (Figure 5), but in general evolutionary rate was similar for homologous traits in different worker castes. We found a complex pattern whereby the evolution of the head shape of major workers was largely decoupled from that of other traits, but was not necessarily evolving faster.

The evolutionary rate of carapace shape was highly divergent across the different parts of the ant (head vs. mesosoma). This observation was most evident with regards to mesosoma shape, which evolved roughly 1.5× faster than head shape (when corrected for variance differences). Moreover, we found that the anterior portion of the head near the mandibles and mouthparts is evolving more quickly than the posterior half. However, there were no significant differences in evolutionary rate among homologous traits between majors and minors. Thus, homologous traits, and not traits within a caste, tended to evolve at similar rates (Figure 1).

The inferred rate similarity among traits does not alone imply the traits themselves are correlated in their evolution (i.e., they could be evolving at similar rates but on different trajectories); thus, we also investigated which sets of traits were correlated during evolution. Here, we found a different pattern, whereby the evolution of mesosoma shape was tightly linked across major and minor workers, but head shape was more decoupled between the two castes. In this way, the head of the major worker was the least integrated with other traits, and the mesosoma of the worker was the most integrated. Previous research in *Pheidole* found that integration among linear measurements was weaker for minor workers than major workers (Pie & Traniello, 2007). In contrast, our analyses found weaker integration between head and mesosoma shape for majors than minors. Thus, no one integration hypothesis was supported—either between homologous traits, or between traits within a caste—but rather a mixture of the two.

The fact that mesosoma shape evolved more rapidly than head shape is somewhat surprising, as the head would presumably be the most related to feeding ecology, a key trait that varies across ant species. One potential explanation is that head shape is under stronger stabilizing selection. However, another potential conclusion is that fast mesosoma evolution reflects relative size and arrangement variation in the underlying muscles that control load carrying and locomotion, which could reflect functional differences in how the ant carries, moves, and performs different tasks. The primary axis of mesosoma variation runs from a stocky shape to a more gracile and elongate one, and most changes are happening repeatedly within limited bounds. There is reason to expect that stocky shapes are common in belowground‐foraging species and that more gracile characteristics are associated with aboveground‐foraging and associated defensive traits like spines (Sarnat et al., 2017; Weiser & Kaspari, 2006). *Pheidole* are known to vary in the extent to which they live and forage in the leaf litter or on vegetation (Mertl et al., 2010), and there could be tradeoffs inherent the designs adapted for moving and foraging on horizontal versus vertical surfaces. This would also explain why major and minor mesosomas are tightly integrated in shape, because they face similar biomechanical challenges due to living and moving in similar environments. Thus, these phenotypes may represent ecomorphs that are repeatedly evolved in each newly colonized region, as in *Anolis* lizards (Mahler et al., 2013). However, given the paucity of behavioral observations for most ant species around the world, further study is required to understand this trait's functional and biomechanical significance. Furthermore, the linking of external geometry with variation in underlying function and performance remains an important avenue for future work on comparative anatomy and biomechanics in ants.

We find support for the hypothesis that the shape of minor and major worker castes can evolve to some extent independently (Holley et al., 2016), promoting the evolution of ecological specialization (Planqué et al., 2016). We emphasize that this is not simply a statement that head shapes are different between majors and minors, which is obvious, but that they can evolve on diverging trajectories (i.e., the major is not just a consistent transformation of the minor). This allows for increased evolutionary “degrees of freedom” in the functional specialization among castes. However, this finding was specific to the head region, as mesosoma shape was tightly integrated across castes. The fact that rates of shape evolution were 1.5 times greater for the highly integrated mesosoma than for the head (Figure 5) suggests that integration in this case does not constrain, but may rather accelerate rates of evolutionary divergence in shape among species (Cheverud, 1995; but see Márquez & Knowles, 2007).

Allometry is a common theme and pattern in development and evolution, and strong relationships between the sizes of different body parts are expected during evolution. Matching this expectation, we found that head and mesosoma sizes were tightly linked both within and among castes (Figure 7). In contrast with the pattern for cranial evolution in birds (Klingenberg & Marugán‐Lobón, 2013), relationships between shape traits and body size were significant, but poorly predictive. While we were not able to account for allometric relationships within species due to our study design, we did find that cross‐species relationships between body size and shape traits were not strong enough to potentially drive other patterns reported in this study. Our estimates of evolutionary rate for size traits showed that the size of each trait evolved faster than its shape (Figure 6), confirming a similar observation by Pie and Tscha (2013). Interestingly, major worker heads evolved at the slowest rate for size and among the slowest for shape despite being the least integrated with other body parts (which should thus release it from constraint by pleiotropic effects; but see Cheverud(1995)). This suggests that this trait is more evolutionarily conserved; future studies investigating the evolutionary consistency of major worker tasks (as in Mertl et al., 2010) and their biomechanical needs would be valuable in explaining this pattern.

In principle, correlations in size and shape among traits/castes could be caused by either selection or developmental constraint. This kind of comparative analysis does not by itself allow for inference of the underlying selective or developmental mechanisms responsible for the patterns of integration that we identify. However, there is a strong body of work on the developmental basis of caste differentiation in *Pheidole*, and especially the role of JH as a developmental switch mediator, that can inform the likelihood of some potential explanations. Notably, classic (Wheeler, 1991; Wheeler & Nijout, 1981, 1983, 1984) and more recent (Rajakumar et al., 2012, 2018) work shows that experimental manipulation of pheromone exposure can alter the relative sizes of *Pheidole* majors and minors, and manipulation of rudimentary wing disks can alter the relative sizes of the head and body (Rajakumar et al., 2018). Moreover, in other insects, it has been shown that relative sizes of different body parts can be experimentally selected for (Frankino et al., 2005; Stillwell et al., 2016). If researchers can manipulate relative size with apparent ease using chemical cues or artificial selection, this implies that evolution may not be constrained from doing the same. We expect that general diversification of body size is likely to due to selection on loci that control body size overall, rather than independent selection on the size of each part. However, the fact that relative sizes of different parts have been maintained in evolutionary time implies selective advantages of the relative sizes of body parts within and among castes (Gould, 1966).

To our knowledge, less is known about the developmental basis of the shape characters we are capturing in our landmark system, so developmental constraints or biases may explain some of the evolutionary correlation in shape we observe. However, the evolutionary modules in the head inferred by our analysis (Figure 4a) do not correspond to the head developmental modules inferred by Yang and Abouheif (2011) in their examination of *Pheidole* gynandromorphs. If both studies are correct, this would imply that developmental modularity does not underlie the macroevolutionary modularity we infer, leaving selection and nongenetic influences, as well as methodological issues with comparing fixed landmarks and semilandmarks, as the most likely explanations for why different regions of the head appear to evolve separately or independently. An interesting future direction would be to attempt to experimentally investigate the developmental bases of the axes of shape variation we identify in our study.

One noticeable feature of the genus *Pheidole'*s global diversification has been the reevolution of similar environmental and behavioral niches in different geographic regions, each radiation following from a single colonization event (Economo, Klimov, et al., 2015; Moreau, 2008). While morphological evolution in this clade has been largely conserved throughout its history (Pie & Traniello, 2007), similar body‐size phenotypes have consistently re‐evolved following each clade's colonization of a new biogeographic realm (Economo, Klimov, et al., 2015). In this study, we observed that New World and Old World radiations of *Pheidole* occupied mostly overlapping portions of morphospace (Figure 3), whereas the Australasian clade occupied a smaller, but still overlapping portion of this same trait space. We found this pattern for size and shape of both head and mesosoma. It remains unclear why some portions of morphospace, and large body size in particular, have not evolved in Australasian taxa. One potential explanation is that niche filling in this most recent radiation is ongoing—indeed, the Australian clade is the youngest of the continental radiations and is still in a more elevated phase of its diversification (Economo et al., 2019).

## 5 CONCLUSION

The morphological and functional differentiation of castes is thought to be a key evolutionary innovation underlying the success of ants and other social insects. Patterns of macroevolutionary integration and modularity within and among castes may provide clues to the selective forces shaping diversification in ants, and the developmental biases and constraints involved in trait divergence (West‐Eberhard, 1979). We find that size evolution is tightly integrated and evolving with homogeneous rates both among parts in a single caste, and across the worker castes. In contrast, our results using geometric morphometric estimates of body shape indicate that while mesosoma shape shows homology integration, head shape has become largely disintegrated between major and minor workers (Figure 3c). Head morphology and its associated musculature are associated with ecological specialization in many taxa, often but not exclusively due to feeding functionality; thus, the differences in head shape between major and minor workers probably represent divergence in their tasks in the colony (Futuyma & Moreno, 1988; Mertl & Traniello, 2009; Smith, 1987). In this case, evolution of developmental pathways facilitating independent evolution of major and minor worker phenotypes could represent key innovations enabling lineages with this trait to occupy multiple specialized strategies at once, or to discover new team strategies emergent from their polymorphism (Anderson & McShea, 2001; Wheeler, 1991; Wheeler & Nijhout, 1981, 1984). Interestingly, the independent evolution of the head does not lead to faster rates of evolution, and in fact, mesosoma shape evolves 1.5× faster than head shape in *Pheidole*. We hypothesize that this rapid evolution of the mesosoma reflects a pattern of frequent adaptation to different biomechanical needs in different microhabitats, but future work is needed to test this hypothesis.

While body‐size polymorphism is a common trait in ants, “complete” polymorphism (i.e., in shape) is rarer but noticeably present in some of the most diverse ant clades (Wills et al., 2018), an observation that hints at a role for polymorphism in adaptability (Wilson, 2003). We propose that, beyond the benefits of body‐size polymorphism, the reduction of morphological integration between distinct behavioral strategies, inclusive of sexes, castes, and alternative reproductive tactics (West‐Eberhard, 1979), could be a recurring key innovation that enables the evolution of adaptive polymorphism and promotes rapid diversification. Further comparative studies on the evolution integration and modularity across radiations of ants with worker polymorphisms, and any concurrent changes in diversification rates and patterns, would be useful for testing this hypothesis.

## CONFLICT OF INTEREST

None declared.

## AUTHOR CONTRIBUTIONS

**Nicholas R. Friedman:** Conceptualization (equal); Data curation (supporting); Formal analysis (lead); Investigation (lead); Methodology (lead); Project administration (equal); Software (lead); Visualization (lead); Writing‐original draft (lead); Writing‐review & editing (lead). **Beatrice Lecroq Bennet:** Conceptualization (supporting); Data curation (lead); Investigation (supporting); Methodology (supporting); Software (supporting); Writing‐review & editing (supporting). **Georg Fischer:** Conceptualization (supporting); Resources (supporting); Visualization (supporting); Writing‐review & editing (supporting). **Eli M. Sarnat:** Conceptualization (supporting); Resources (supporting); Visualization (supporting); Writing‐review & editing (supporting). **Jen‐Pan Huang:** Formal analysis (supporting); Resources (supporting); Writing‐review & editing (supporting). **L. Lacey Knowles:** Conceptualization (supporting); Funding acquisition (equal); Project administration (supporting); Resources (supporting); Supervision (supporting); Writing‐review & editing (supporting). **Evan P. Economo:** Conceptualization (equal); Funding acquisition (equal); Project administration (lead); Supervision (lead); Visualization (supporting); Writing‐review & editing (equal).

### OPEN RESEARCH BADGES

This article has earned an Open Data Badge for making publicly available the digitally‐shareable data necessary to reproduce the reported results. The data is available at https://doi.org/10.5061/dryad.gqnk98sjx.

## Supporting information

Appendices S1 and S2

## Data Availability

Data for this paper can be accessed on Dryad at https://doi.org/10.5061/dryad.gqnk98sjx.
