# Supplementary material for: Macroevolutionary integration of phenotypes within and across ant worker castes
Source: Ecol Evol. 2020 Aug 18;10(17):9371–83. doi: 10.1002/ece3.6623 (PMC7487254; doi:10.1002/ece3.6623)
Supplement: Supplementary file 1 — Appendices S1 and S2 [file ECE3-10-9371-s001.docx]

## Appendix 1: Landmark definitions for *Pheidole* head.

| **Number** | **Type** | **Definition** |
| --- | --- | --- |
| **1** | Fixed | Left eye – Posterior apex |
| **2** | Fixed | Left eye – Anterior apex |
| **3** | Fixed | Right eye – Posterior apex |
| **4** | Fixed | Left eye – Anterior apex |
| **5** | Fixed | Clypeal median – Posterior margin apex |
| **6** | Fixed | Clypeal median – Anterior margin apex |
| **7** | Fixed | Left mandible intersection with lateral clypeal margin |
| **8** | Fixed | Right mandible intersection with lateral clypeal margin |
| **9** | Fixed | Left antenna torulus – Anterior margin apex |
| **10** | Fixed | Right antenna torulus – Anterior margin apex |
| **11** | Fixed | Occipital center – Posterior Apex |
| **12 – 19** | Semi | Seven equidistant points along left occipital margin between landmarks 3 and 1, with right side removed |
| **20 – 25** | Semi | Reflection of points 12 – 19. |

## Appendix 2: Landmark definitions for *Pheidole* mesosoma.

| **Number** | **Type** | **Definition** |
| --- | --- | --- |
| **1** | Fixed | Propodeal insertion of petiole – Superior |
| **2** | Fixed | Propodeal insertion of petiole – Inferior |
| **3** | Fixed | Propodeal spiracle |
| **4** | Fixed | Intersection of mesopleural, propodeal margins – Inferior apex |
| **5** | Fixed | Intersection of pronotal, mesopleural margins – Inferior apex |
| **6** | Fixed | Intersection of mesopleural, propodeal margins – Superior apex |
